# Acceptability to donate human milk among postnatal mothers at St. Francis hospital Nsambya, Uganda: a mixed method study

**DOI:** 10.1186/s13006-024-00615-2

**Published:** 2024-02-01

**Authors:** Mohammed A. M. Ahmed, Charles Patrick Namisi, Nakibuuka Victoria Kirabira, Micheal Webba Lwetabe, Joseph Rujumba

**Affiliations:** 1 Department of Paediatrics, Faculty of Medicine and Surgery, Mogadishu University, Mogadishu, Somalia; 2 Department of Paediatric Cardiology, Heart Institute, Mulago complex, Kampala, Uganda; 3 Mother Kevin Postgraduate Medical School, Uganda Martyrs University, Kampala, Uganda; 4 St. Francis Hospital Nsambya, Kampala, Uganda; 5 Department of Paediatrics and Child Health, College of Health Sciences, Makerere University, Kampala, Uganda

**Keywords:** Donor Human milk (DHM), Donated breast milk, Wet nursing, St. Francis Hospital Nsambya

## Abstract

**Background:**

The World Health Organization recommends the use of donated human milk (HM) as the second-best option for mothers who are temporarily unable to provide sufficient breast milk to meet the needs of their infants. However, HM donation is yet to become an accepted practice in Uganda. We assessed the level of, and factors associated with acceptability to donate HM among postnatal mothers at St. Francis Hospital Nsambya (SFHN).

**Methods:**

A cross-sectional sequential explanatory mixed method study was conducted between October 2018 and March 2019. A questionnaire on sociodemography, awareness and likely acceptability to donate HM was administered to 410 postnatal mothers at SFHN. Multivariate logistic regression analysis was undertaken to determine factors associated with acceptance of HM donation. Focus Group Discussions (4) and Key Informants Interviews (4) were used to explore factors influencing behaviours to donate human milk. Qualitative data were analysed using a content thematic approach.

**Results:**

Overall acceptance of donating the HM was 77.6%, and the significant factors were: willingness to express the HM (AOR 7.5; 95% CI 3.01, 18.68); wet-nursing knowledge (AOR 2.3; 95% CI 1.1, 5.0) and visit to under-5 years’ clinic (AOR 21.3; 95% CI 2.3, 196.9). The major themes in relation to accepting to donate HM were wet nursing experience, and confidence in donating the HM, and its perceived effectiveness. There were concerns about the safety and adequacy of HM and fear of transmitting criminal behaviours and mental illness through human milk.

**Conclusions:**

Acceptance to donate HM among postnatal mothers at St. Francis Hospital Nsambya was very high. Willingness to express and store human milk, prior knowledge about wet nursing and a visit to an under-five outpatient clinic were associated with acceptance. Thus, establishing a human milk bank is feasible in the study setting.

## Background

Early breastfeeding is critical to a child’s overall health as it affects immune system development and maturation [1, 2]. In Uganda, 91% of all newborn infants are breastfed within the first one hour of life [3]. Breastfed preterm infants have been observed to display less feeding intolerance, develop fewer severe infections, less necrotizing enter colitis (NEC), and experience decreased lengths of hospital stay [4]. According to the World Health Organization, preterm and low birthweight (LBW) infants should be fed on mother’s own milk, and, where not possible, use of pasteurized donor human milk is recommended [5]. The first method used to address breast milk shortages was the “wet nursing method”, and later, “formal human milk banking” was introduced [6] to use pasteurized donor human milk (PDHM) as a standard. As of 2016, over 500 human milk banks had been established, with most being in Europe and the USA. There are two hundred and six in Europe, forty-four in Asia, four in Australia and seventy in Africa. Of those in Africa, sixty are in South Africa, six are in Cameroon, one was planned in Kenya and one is in Nigeria [7]. Since then, human milk banks have been growing globally and had increased to 756 in 66 countries in 2020 [8, 9]. There is wide variation in the acceptance of HM worldwide, ranging from 11% in Nigeria to 84% in India. Several factors have been identified to be associated with donating human milk. In Ethiopia, the acceptance of donor milk was more likely among mothers who had previously heard about donor milk banking, wet nursing and those mothers who had visited a neonatal intensive care unit [10]. Human milk donation is more likely to happen if there is encouragement of human milk donation by healthcare professionals, receiving information on breast milk expression by the healthcare unit, and getting help from the health professionals to breastfeed. Admission of newborns to the neonatal unit has been associated with a lower prevalence of HM donation. Other reasons for not accepting DHM include; fear of transmitting diseases (28%), fear of transfer of genetic traits (22%) and religious and cultural taboos (14%) [11]. In addition, there were concerns about the safety of donor human milk and discomfort about using another mother’s milk. Participants believed that education on the importance of human milk and transparency on the processes involved in sourcing and preparing donor milk would improve the acceptability [12]. At St. Francis Hospital Nsambya, donated human milk for preterm babies has been in use since 2016, where mothers with an excess of milk were identified and basic screening investigations like HIV, Hepatitis B, and syphilis were done, and then their milk would be pasteurized using the Pretoria method. Pretoria Pasteurization involved boiling the pot of water, then taking it off the heat, a covered jar of expressed milk is then placed in the hot water for 20 min, removed and allowed to cool before use [13]. The use of human milk exclusively for preterm babies reduced the incidence of NEC dramatically. With the increasing number of preterm babies, the current supply of DHM is not enough to meet the needs. In addition, to optimize breast milk production for mothers at the hospital, educational programmes on breastfeeding were done during antenatal and postnatal visits. Furthermore, all mothers were supported by midwives on the ward to express breast milk into a bottle for their babies and to ensure that by the time of discharge from hospital, these babies have had a milk feed. Despite all these efforts sometimes mothers with preterm babies had no milk available within the first 48 h. Therefore, to ascertain more information about human milk donation, this study assessed the level of acceptance to donate milk, as well as the barriers and enablers for human milk donation. The findings of this study will be used to influence the process of human milk donation for a future human milk banking and to support and improve breastfeeding practices at St. Francis Hospital Nsambya.

## Methods

### Study design

This was a cross-sectional, mixed methods study conducted at St. Francis Hospital Nsambya between October 2018 and March 2019. The quantitative study assessed the level of acceptance and factors associated with human milk donation, while the qualitative part of the study explored enablers and barriers that influence acceptance to donate human milk.

### Study setting

St. Francis Hospital Nsambya is a 361 bed capacity, Private-Not-For-Profit, tertiary and teaching hospital located in Makindye division of Kampala Capital City. The hospital provides both inpatient and outpatient services. St. Francis Nsambya Hospital has an approximate catchment population of 250,000, with approximately 14,000 admissions and antenatal attendances of 22,000 per year (Hospital records 2018). Approximately 400–500 deliveries are conducted per month, which totals to 5500 deliveries annually [14]. The Paediatrics department offers neonatal services, including a neonatal intensive care unit (NICU) with 10 beds and a baby unit of approximately 40 beds, as well as outpatient clinics, including preterm, neurodevelopmental, neurology, chest and general paediatrics. Nsambya Hospital has a Baby Friendly Hospital committee and this meets once a month whose basic aim is to promote and protect breastfeeding. Additionally, breastfeeding education is done by the midwives in the antenatal clinics and the postnatal ward. Lactation support is offered to postnatal mothers and those in the neonatal unit. Despite these efforts, the volume of donated human milk remains inadequate to match the number of preterm babies, hence the idea to establish a Human Milk Bank (HMB). For the past two years, the hospital has been conducting activities to promote community awareness about the need for human milk donation as part of the hospital plan to establish a human milk bank. Such activities included community education through radio, television talk shows, charity walks during celebrations to mark the World Prematurity Day, health education talks in the ANC, immunization units and postnatal wards.

### Study population

Postnatal breastfeeding mothers aged 18 years and above in postnatal wards and department of Paediatrics, St Francis Hospital Nsambya.

#### Inclusion criteria

All postnatal mothers above 18 years of age attending postnatal care, immunizations, and departments of paediatrics, or who were referred from other health facilities to St. Francis Hospital Nsambya during the study period who had consented, were included.

#### Exclusion criteria

Critically ill mothers who were not able to answer survey questions and those mothers whose babies had died.

### Sample size determination

#### Sample size for quantitative phase

The sample size for this study was 410 postnatal mothers calculated based on Kish Leisle [15] using a single population proportion formula with a 95% confident interval. The percentage of human milk donation acceptance among Nigerian women was 39.9% [16], with precision set at 5%, and a 10% non-response was estimated.

For the qualitative component of the study, thirty participants were grouped into four focus group discussions (FGDs), each having a minimum of seven and a maximum of eight mothers. In addition, there were four key informant interviews (KIIs) that were conducted with health workers involved in the care of preterm babies and mothers who had donated human milk.

### Data collection methods and tools

The study began with providing information about the research to postnatal mothers who were attending the postnatal clinic and department of paediatrics at St. Francis Hospital Nsambya. Those who were willing to participate in the study were then requested to undergo a screening process. The research assistant obtained informed consent from mothers who met the inclusion criteria. This was followed by collecting data on sociodemographic characteristics, health service characteristics, and sociocultural factors using a pretested interview questionnaire that also contained questions on the acceptance to donate human milk.

For the qualitative data, the composition of the FGDs was based on the quantitative findings, where mothers who had attended the Outpatient Department (OPD) were more likely to accept human milk donation. Thus, we conducted two FGDs each for mothers receiving inpatient services (NICU and baby unit) and mothers receiving outpatient services (recruited from postnatal care, immunization and paediatric clinic). The FGDs lasted 1–2 h. The key informant interviews were purposively selected based on their knowledge and experience of human milk donation. The study team introduced themselves, explained the purpose of the study and obtained written consent from all participants. Trained qualitative researchers moderated the discussions while the principal investigator audio recorded the proceedings and took notes.

### Data quality

A structured interview questionnaire was developed in English, then translated into the local language, Luganda, and was translated back into English to ensure consistency. The questionnaire was pretested on 5% (5%) of the total sample size at the study site. These data were not included in the final analysis. Data were collected by trained research assistants. A two-day intensive training was held for all research assistants on the content, objectives, data collection tools and how to interview the study subjects. At the end of each working day, the principal investigator and research assistants reviewed the collected data for accuracy and completeness.

For qualitative data collection, interviews were undertaken in either the Luganda or English language using a semi-structured interview guide. The interviews were conducted in a well-lit, private room that was located in the paediatric outpatient department. A semi-structured interview guide contained questions selected based on a review of the literature. The interview guides contained open response format questions that enabled probing to clarify responses. The FGDs and Key Informant Interviews (KIIs) were facilitated by the study team members with training and experience in qualitative research methods. To ensure standardization, all questions on the guide were asked. All interviews and discussions were audio recorded and transcribed by two independent research assistants fluent in both languages. Those done in Luganda, transcripts were later translated to English.

### Data analysis

Data were captured in EpiInfo database and exported into STATA version 13.0 software (STATA Corporation, Houston, Texas) for analysis. Statistical analysis was performed using a three-stage method, univariate, bivariate and multivariate, respectively. In the univariate analysis, continuous variables were summarized using means and standard deviations, and categorical variables were summarized using frequencies and proportions. In the bivariate analysis, chi-square tests were used to examine the relationships between the independent and dependent variables. Factors associated with a *p* - value of < 0.05 were considered statistically significant. All factors with a *p* - value of 0.25 were included in the multivariate logistic regression analysis. Multivariate logistic regression analysis was used to determine the adjusted odds ratio and factors associated with acceptance to donate human milk for banking. We considered backward stepwise elimination in the multivariate logistic regression analysis to identify factors associated with acceptance at a *p* - value < 0.05.

For qualitative purposes, recorded data were transcribed verbatim by a qualitative research assistant. A second independent research assistant also transcribed and translated the transcripts to ensure validity. Both transcriptions were compared for discrepancies. The transcriptions were analysed and coded by using a content thematic approach [17], taking into account both manifest and latent content to determine themes and sub-themes of acceptability for donating milk. The principal investigator (PI) and another qualitative researcher coded the transcripts independently to enhance consistency. The researcher and the supervisors jointly compared and finalized the themes and labelled them. The analysis draws on a theoretical framework of acceptability of health care interventions [18]. The key constructs of the framework are; (1) affective attitude (how an individual feels about the intervention in this case donation of HM), (2) burden (perceived effort required to participate in the intervention), (3) ethicality (intervention’s goodness of fit with individual’s value system), (4) intervention coherence (participant’s understanding of how intervention works), (5) opportunity costs (benefits, profits and values to be given up to engage in the intervention), (6) perceived effectiveness (perception that intervention has achieved purpose), and (7) self-efficacy (participant’s confidence that they can perform the intervention) [18]. Perspectives of mothers were triangulated with those of healthcare workers. Selected quotations from study participants were used in the presentation of the study results.

### Study variables

Dependent variable: Acceptance of the donation of human milk for banking.

Independent variables: Postnatal maternal factors, sociocultural factors of postnatal mothers.

Mediating variables: Health system factors.

#### Operational definitions

Acceptance of human milk donation: Refers to mother’s willingness to donate human milk for banking [19].

Human Milk Banking (HMB): Refers to services which collect, screen, process, and dispense human milk to hospitals or recipients [20].

Awareness of mothers about donor human milk banking: A mother was considered aware about donor human milk banking if she had ever heard about donor milk banking [21].

Human milk donation: Refers to the act of a lactating mother giving breast milk for human milk banking [21].

Wet-nurse: Refers to a mother who breastfeeds another woman’s baby [22].

The postnatal period: Refers to the first six weeks after child birth [19].

The postnatal mother: Refers to a mother who had given birth to a baby with in the last six weeks [19].

## Results

### Sociodemographic and maternal characteristics of the participants

The study was conducted from October 2018 to March 2019, and we studied 410 postnatal mothers. Their median age was 29 years (IQR: 26, 32), of which 68.8% were aged between 25 and 34 years and 15.1% were 35 years or older. The majority (62.4%) of the respondents were university graduates, 75.6% of the women were employed, and 86.6% of them had attended at least four ANC visits. During their previous pregnancy, 54.6% of the respondents had attended only one postnatal clinic (PNC) visit, and the remaining had attended two or more PNC visits. (Table 1)

**Table 1:** Sociodemographic and maternal characteristics of the participants *N* = 410

Variable	Freq	(%)
Age		
Mean(SD)	29	49.3
Age groups		
< 25	66	16.1
25–34	282	68.8
35 + yrs	62	15.2
Level of Education		
Non-graduates	154	37.6
Graduates	256	62.4
Participant occupation		
Unemployed	100	24.4
Employed	310	75.6
Participant religion		
Christians	345	84.1
Muslim	65	15.9
Marital Status		
Unmarried	152	37.1
Married	258	62.9
Husband education *n* = 406		
Non-graduates	100	24.6
Graduates	306	75.4
Household monthly Income		
< 500,000	133	32.4
> 500,000	277	67.6
Number of pregnancies carried		
< 1 pregnancy	118	28.8
1 + Pregnancies	292	71.2
Parity		
<=3	322	78.5
4+	88	21.5
ANC previous pregnancy		
Not applicable	159	38.8
<=3 ANC	23	5.6
4 + ANC	228	55.6
ANC last pregnancy(current baby)		
<=3 ANC	55	13.4
4 + ANCs	355	86.6
Participant has ever received breastfeeding counselling *n* = 402		
No	128	31.8
Yes	274	68.1
PNC visit previous pregnancy		
Not pregnant	130	31.7
1 Visit	224	54.6
2 + visits	56	13.7

### Acceptance to donate human milk

Of the 410 postnatal mothers interviewed, 77.6% (95% CI 73.5, -81.6) were willing to donate human milk, and the most common reason to donate was if they had excess breast milk (66%). 63% were willing to donate to help infants in need, and 55% were willing to support another mother who lacked breast milk (Table 2). On the preference to donate human milk, 58.1% of the respondent’s preferred donation to a relative, while on receiving donated human milk, 94.6% of the respondents were not restricted to a relative. (Table 2)

**Table 2:** Level of acceptance and reasons for donating human milk

Variable	Freq	(%)
Acceptance to donate human milk *n* = 410		
Yes	318	77.6
No	92	22.4
Reasons for accepting to donate DHM *n* = 318		
Excess breast milk	208	65.4
Help infant in need	197	62.1
Support another mother	172	54.1
Breastmilk better for growth	42	13.2
Best food for infants	40	12.6
Human breastmilk prevents diseases	35	11.0
Expensive formula milk	14	4.4
For health promotion	5	1.6
Acceptance to use donated human milk		
No	129	31.5
Yes	281	68.5
Reasons for accepting to use DHM *n* = 281		
Best food for infants	182	64.8
Breast milk better for growth	174	61.9
Breast milk prevents diseases	62	22.1
Help infant in need	20	7.1
Expensive formula milk	18	6.4
Reason for not accepting to use DHBM *n* = 129		
Fear of disease transmission	79	61.2
Fear unhygienic milk	47	36.4
Idea not liked	44	34.1
Fear genetic mixing	18	14.0
Spouse/family not supportive	9	7.0
Not accepted culturally	4	3.1
Religious issues	2	1.6
Infant formula preference	2	1.6
Preference to donate human milk		
Not relative	172	42.0
Relative	238	58.1
Preference to receive human milk		
Not relative	388	94.6
Relative	22	5.4

### Reasons for accepting to donate human milk

The most common reasons for accepting to donate among postnatal mothers were having excess breast milk, followed by helping infants in need and supporting mothers who lacked breast milk (Table 2). While fear of disease transmission, anxiety about safety, and genetic mixing were the major reasons for not accepting the use of donated human milk, others preferred not to donate due to non-supportive environments and cultural and religious issues (Table 2).

### Willingness to receive donor human milk for own infant

When asked about the acceptance to use donated human milk, of the 410 mothers interviewed, 69.4% were willing to use and 31.5% were not willing to use donated human milk. Among those who were willing to use donated human milk 65.0% said it was because human milk is the best food for infants, followed by human milk is better for growth by 62.0%, and 22.0% was because human milk prevents diseases. Of the 129 (31.5%) mothers who were not willing to use donated human milk, the most common reason for decline was fear of disease transmission (61.2%), a perception that donated milk is unhygienic (36.4%) and not accepted culturally (3.1%) (Table 2).

### Participants’ awareness of human milk donation

Regarding awareness about wet nursing, 79.3% of the respondents had ever heard about wet nursing, and 50.2% of the respondents were aware of human milk banking. A total of 87.1% of mothers in the study had never breastfed another person’s child. A total of 89.0% agreed that collecting and storing human milk was useful. A total of 68.3% of the respondents had ever visited a clinic for under-five children.

### Factors associated with acceptance to donate human milk at St. Francis Hospital Nsambya

In bivariate analysis, acceptance of donating human milk was affected by women’s level of education. Women who were graduates had an OR of 1.94 (95% CI 1.22, 3.11) times more likely to accept human milk donation than non-graduates. Acceptance to donate human milk was also affected by the level of education of women’s husbands, with women who had graduate husbands being 1.8 (95% CI 1.09, 3.00) times more likely to accept to donate milk. Women who had heard about wet nursing were 2 (95% CI 1.18, 3.40) times more likely to accept human milk donation than those who had not heard about wet nursing. However, women who believed that collecting and storing human milk was useful were 6.28 (95% CI 3.15, 12.52) times more likely to accept human milk donation (Table 3).

**Table 3:** Factors associated with acceptance to donate human milk at St. Francis Hospital Nsambya

	Acceptance to donate		Bivariate	
Participant Characteristics	No number (%)	Yes number (%)	cOR (95% CI)	*p*-value
Age group < 25	14 (15.2)	52 (16.4)	1	
25–34	63 (68.5)	219 (68.9)	0.94 (0.49, 1.80)	0.842
35 + yrs	15 (16.3)	47 (14.8)	0.84 (0.368, 1.93)	0.687
Level of education				
Non-graduates	46 (50)	108 (34)	1	
Graduates	46 (50)	210 (66)	1.94 (1.21, 3.11)	0.006
Husband education				
Non-graduates	31 (33.7)	69 (22)	1	
Graduates	61 (66.3)	245 (78)	1.8 (1.09, 3.00)	0.023
Parity				
<=3	70 (76.1)	252 (79.3)	1	
4+	22 (23.9)	66 (20.7)	0.78 (0.46, -1.33)	0.364
ANC current pregnancy				
<=3 ANC	15 (16.3)	40 (12.6)	1	
4 + ANCs	77 (83.7)	278 (87.4)	1.35 (0.71, 2.58)	0.357
PNC visit after last pregnancy				
2 + visits	16 (17.4)	40 (12.6)	1	
1 Visit	52 (56.5)	172 (54.1)	2.42 (1.05, 5.61)	0.038
Not pregnant	24 (26.1)	106 (33.3)	3.31 (1.38,7.95)	0.007
Ever visited under-five OPD wards				
No	32 (36.8)	87 (30.2)	1	
Yes	55 (63.2)	201 (69.8)	1.34 (0.81, 2.22)	0.249
Counselled on breastfeeding at under-five OPD				
No	31 (35.6)	90 (31)	1	
Yes	56 (64.4)	200 (69)	1.23 (0.74, 2.04)	0.421
Experienced breastfeeding difficulty?				
No	69 (78.4)	247 (82.1)	1	
Yes	19 (21.6)	54 (17.9)	0.79 (0.44,1.43)	0.441
Acceptance to use donated human milk				
No	87 (94.6)	301 (94.7)	1	
Yes	5 (5.4)	17 (5.3)	0.98 (0.35, 2.74)	0.973
Heard about wet-nursing?				
No	28 (30.4)	57 (17.9)	1	
Yes	64 (69.6)	261 (82.1)	2 (1.18, 3.40)	0.010
Heard about human milk banking?				
No	52 (56.5)	152 (47.8)	1	
Yes	40 (43.5)	166 (52.2)	1.42 (0.89, 2.27)	0.142
Have you ever breastfed another mother’s child?				
No	80 (87)	277 (87.1)	1	
Yes	3 (3.3)	22 (6.9)	2.12 (0.618, 7.26)	0.232
Is collecting and storing human milk useful?				
No	23 (25)	16 (5)	1	
Yes	68 (74)	297 (93.4)	6.28 (3.15, 12.52)	< 0.001

In multivariate analysis, having ever visited the under-five OPD clinic 21.29 (2.30, 196.9), having heard about wet nursing 2.33 (1.10, 4.96), and being one who perceived collecting and storing human milk as useful 7.51 (3.01, 18.68) were independently associated with accepting donation of human milk (Table 4).

**Table 4:** Factors associated with acceptance to donate human milk at St. Francis hospital Nsambya

Variable	Bivariate		Multivariate	
Age group	cOR (95% CI)	*p*-value	aOR (95% CI)	*p*-value
< 25	1		1	
25–34	0.94 (0.49, 1.80)	0.842	0.63 (0.22, 1.82)	0.397
35 + yrs	0.84 (0.37, 1.93)	0.687	0.44 (0.11, 1.77)	0.250
Level of education				
Non-graduates	1		1	
Graduates	1.94 (1.22, 3.11)	0.006	1.30 (0.62, 2.73)	0.482
Husband education				
Non-graduates	1		1	
Graduates	1.8 (1.09,3.00)	0.023	1.35 (0.60, 3.05)	0.463
Ever visited under-five OPD wards?				
No	1		1	
Yes	1.34 (0.81, 2.22)	0.249	21.29 (2.30, 196.9)	**0.007**
Heard about wet-nursing?				
No	1		1	
Yes	2 (1.18, 3.40)	0.010	2.33 (1.10, 4.96)	**0.028**
Is collecting and storing human milk useful?				
No	1		1	
Yes	6.28 (3.15, 12.52)	0.000	7.51 (3.01, 18.68)	**< 0.001**

#### Qualitative results

Most postnatal mothers in the qualitative component of the study were aged 25–34 years, more than half of the mothers had attained tertiary education, and almost all of them were employed (Table 5).

**Table 5:** Characteristics of mothers in the FGDs and KII

Characteristics	Number
Age range (yrs)	
18–24	6
25–34	24
35 + yrs	4
Education level	
Graduate	18
Non-graduate	16
Employment status	
Employed	26
Un-employed	8
Participant categories	
FGD at IPD	# mothers
First FGD	8
Second FGD	7
FGD at OPD	
First FGD	7
Second FGD	8
KII	4

KII- Key Informant Interviews, FGD-Focus Group Discussion, IPD-Inpatient Department, OPD Outpatient Department

### Summary of quantitative results

The qualitative data explored reasons for acceptance to donate human milk. The results are presented using a Theoretical Framework of Acceptability of Health Interventions [18] and end with recommendations from participants. (Table 6).

**Table 6:** Summary of results from the acceptability to donate human milk study, reported using constructs from the theoretical framework of acceptability (TFA) by Sekhon et al.

TFA construct	Definition	Sub themes	Major themes	Suggestions
Affective attitude	Affective attitude implies how an individual feels about the intervention	Breast milk best food/no substitutes Breast milk is irreplaceable Self-relief from pain or congestion	Enablers of human milk donation	Build on the enablers to educate and sensitize people about human milk donation
Self-efficacy	Participant’s confidence that they can perform behaviour required by intervention	Would rather donate breast milk than discard it		
Intervention coherence	Extent to which the participant understands the intervention and how it works	*‘I have seen babies wet nursed grow well’* Breastfed babies grow healthy Positive experience with donated breast milk		
Perceived effectiveness	Extent to which intervention is perceived to achieve its purpose	Breast milk saves lives/ especially of premature		
Burden	The perceived amount of effort required to participate in the intervention	May not have time to donate	Barriers of human milk donation	Process of human milk donation should be quick Consider pick up services from home Doctors to assess those suitable to donate Community education about human milk donation to address those fears. Provide health education & counselling Involvement of male partner in education and decision making in human milk donation
Opportunity costs	Extent to which benefits, profits, or values must be given up to engage in the intervention	Fear the milk may not be enough for own baby May lead to distorted body image /breast sagging Fear to test for HIV and know one’s sero-status Fear to transmit infections		
Ethicality	Extent to which the intervention has good fit with an individual’s value system	Negative socio-cultural beliefs (a belief that criminality and mental illness can be transmitted through donated human milk & fear that mother can be harmed through donated human milk) Partner/spouse refusal		

#### Enablers of human milk donation

##### Human milk best food/no substitutes

It was agreed among the FGD participants and KIIs that there was no substitute for human milk for a newborn baby, whether preterm or full term. They noted that mothers who did not breastfeed their babies were denying them the best quality food and that it would interfere with the proper development of their children. As one of the mothers stated,

“*Breast milk is well balanced with all nutrients whatever the mother eats the baby will get it through breastfeeding*”. *(R1/FGD2, 31 years old, a mother of 3 children).*

Another mother described human milk as irreplaceable:

*“However expensive the formula can be it is not a replacement to breast milk. Breast milk is very important to baby”. (R6/FGD1, a 25-year-old mother).*

A health professional also disagreed with the idea of mothers introducing formula milk to their newborn babies, as this could harm their babies. She considered formula milk to be a risk to newborns, especially preterm babies, as it may expose them to health complications.

***“...****Nothing else other than breast milk their intestines can’t digest what you are giving them. So they will swell the tummies once you give them formulas.” (KII1 NICU nurse, a mother and frequent human milk donor at St. Francis Hospital Nsambya).*

#### Self-relief from pain or congestion

Some participants also considered human milk donation as a way of reducing congestion of breast. They looked at it as a relief for the mother who is experiencing painful breasts due to milk engorgement, in general, to avoid breast milk engorgement mothers were offered counselling to early initiation and regular breastfeeding, warm/cold compress, as well as hand expression or using pump when they cannot nurse.

*“I can donate if I have enough to avoid the pain and swelling of the chest*”. *(R5/FGD2, 25-year-old mother of 3 children).*

“*Most people have much milk like me I have excess and wet my blouses.. so I will donate*”. *(R3/FGD2, 25-year-old first-time mother).*

While some mothers looked at it as a way of regulating breast milk flow to avoid choking for their own babies, especially for the mothers who have a lot of breast milk

“*You would be breastfeeding your baby and then you see the milk choking him/her that shows you that you have excess so to reduce it is better option when you donate to needy preterm*”. (*R1/FGD2, a 31-year-old mother of 3 children).*

#### Would rather donate human milk than discard it

Most of the participants demonstrated confidence that they could donate if they had excess human milk, especially because the preterm infants were unlikely to survive without human milk. They considered human milk donation as an act of kindness and a sacrifice to save a life.

*“Really when I have excess and there is another mother who doesn’t have I would rather donate it to them than to flash it out. And some can’t even afford to buy the formula so there is need to really donate this milk to the needy mothers and preterms”. (R5/FGD4, a 30-year-old mother of 3 children).*

Some participants attributed the willingness to donate human milk to having a humane and motherly spirit and understanding that anyone can be in need anytime.

*“If you have a motherly spirit you will donate because these things are like this today with another person tomorrow it will be you crying and looking for a donor to bless your child. So it is good to donate and I think majority women will be willing to donate”. (R6/FGD4, 28-year-old mother of 3 children).*

#### Seeing children grow on another woman’s milk *(wet nursing)*

Most women who had seen babies wet-nursed grow well or had heard about wet nursing were willing to donate human milk, as one of the participants explained:

*“I had my friend and her sister went abroad for greener pastures, she left a baby at 2 months and her sister used to breastfeed two babies and they grew as if they were twins and they are 3 years.. Wet nursing should be stopped completely and we resort to breast milk donation because the mother will have been tested very well at the hospital by the doctors.” (R2/FGD3, 28-year-old first-time mother).*

Emerging from the above narrative is the appreciation that human milk donation can help to overcome the limitations of wet nursing, mainly the spread of infections and diseases to the baby.

*“I think wet nursing is not good the world has changed, and many women are sick with HIV and other diseases. It is not good it is better to donate because it is safe they test the donors and breast milk is kept safely”. (R4/FGD1, 23 years old, first-time mother).*

#### Positive experience with donated human milk

A few of the mothers had used donated human milk and described how human milk donation worked for babies, as one of them narrated.

*“I have gone through a lot myself as a mother of a preterm when they told me to express milk, but I totally had nothing until a nursing sister helped me. So I will be willing to help another mother I have learnt the hard way.. I later saw my baby responding so well and looking better every other day until I got my own breast milk. So I can be willing to donate having learnt that helping is good and nothing bad another person’s breast milk can do to your baby once tested. (R4/FGD3, a 28-year-old first-time mother).*

Other participants believed that children grow healthy and develop immunity through human milk, as a mother explained:

*“Children grow healthy, children gain resistance to diseases when they breastfeed there is good immunity for the breastfed baby”. (R1/FGD3, 34-year-old mother).*

Another mother also related children’s education performance to human milk, where she said:

*“I think the babies should breastfeed because the human milk is good, and they tell us that we should breastfeed our babies well. I have 4 kids they are looking so healthy because of*.

*breastfeeding and are brilliant in class”*. *(R8/FGD3, 33-year-old mother of 4 children).*

### Human milk saves lives, especially of premature

A great number of respondents considered human milk donation as an act of saving the lives of the pre-matures. They all agreed that the greatest motivation for them to donate human milk was because they knew that they were saving a life, as these preterm babies could only survive on breast milk, as a 26-year-old first-time mother said:

“.. *So you need to be determined to do it [human milk donation] and save a life*.” *(R1/FGD3, 34-year-old mother).*

A staff member and a mother also described the importance of human milk, and by seeing the vulnerable babies, you will need to do good saying:

*“But after learning about the superiority of breast milk over the formula, then you get a heart to help someone who lacks to save a life”. (KII2, a doctor at St. Francis Hospital Nsambya and a mother who ever donated).*

Another healthcare professional stated that by human milk donation, preterm infants can survive and cannot tolerate formula.

*“I am a mother so seeing someone’s babies die and also a healthy worker taking care of those babies I knew what could help those babies to survive. I knew what they could feed on and survive once such preterm babies are put on formula milk they will die”. (KII1 NICU nurse, a mother and frequent human milk donor at St. Francis Hospital Nsambya).*

### Barriers to human milk donation

The most common barriers to accepting human milk donation among participants were fear of not having time, breast milk may not be enough for one’s own baby, loss of body image, fear of testing for HIV and knowing one’s HIV status, and negative social cultural beliefs.

### Mother may not have time to donate

Participants agreed that although most of them would be willing to donate, they were afraid that they may not have the time to come to the hospital to do it because of competing responsibilities. Others wondered if there would be mechanisms for collecting donated human milk from home to relieve the donors the time of moving to the hospital.

“*Some will be willing to donate but time. Some mothers are too busy to come to hospital to donate”. (R6/FGD2, a 25-year-old of 3 children).*

Some participants who are working suggested that it would be time saving if the milk could be collected from their places of work.

“*To add on that we working mothers we have no time you tend to forget your baby*”. (*R4/FGD1, 23-year-old first-time mother).*

### Fear the milk may not be enough for own baby

Some of the participants were afraid of not having enough breast milk for their own babies if they decided to donate. They looked at this as depriving their own babies at the expense of those who needed the milk as one mother noted.

*“You might donate and then you end up lacking enough for your baby. There was a time I wanted to donate for a lady in ICU and before a short while something came up and stressed me and pressure short up and my breast milk disappeared for a whole day and a half.. So for me I have that worry as you can donate but fail to produce enough for your baby”. (R3/FGD1, a 33-year-old mother with 3 children).*

### May lead distorted body image /breast sagging

The worry of women losing their body image as a result of donating human milk was mentioned in all four group discussions. The participants feared that pressing or pumping their breasts to produce milk would cause their breasts to sag and their bodies to lose shape, yet this was a major concern to most women.

“*Some people will wonder how they can breastfeed their babies as well donate, will they fear losing weight and being bum less and breasts grow floppy. So it might not work well.” (R2/FGD2, a 19-year-old first-time mother).*

Other participants further noted that sagging breasts was not only a concern for the women but also for their husbands, and most men preferred to have women with firm breasts.

*“Our spouses also don’t like floppy boobs (breasts) they keep looking for young shooting boobs. That is why women breastfeed for a short while and then put them on the formula due to the demands of men”. (R7/FGD1, 23 year old, a mother of 2 children).*

The above narratives reflect a misconception that breastfeeding causes breasts to sag and efforts to promote breastfeeding and human milk donation should address this misconception.

Some study participants argued that having a human milk bank will be a means for women to escape breastfeeding and opt to buy milk just to keep their breasts firm.

*“.. While other say women will totally stop breastfeeding their children totally in the guise that there is free breast milk in the breast milk bank, they can buy or follow all procedures to get it. That they would rather keep their boobs looking good and nice than breastfeeding babies and they look old”. (R2/FGD2, 19-year-old first-time mother).*

Implied in the above narrative is the potential for the likely misuse of donated human milk and requires strict guidelines to attain the intended goal.

### Fear of testing for HIV and knowing one’s sero-status/fear of transmitting infections

One of the greatest fears that all the participants mentioned was the fear of testing or being tested for HIV before human milk donation. This was a concern that potential donors would be afraid of being tested for HIV since it is a requirement for one to donate

“*Most young mothers don’t like to test and know their sero statuses. Instead, they worry much and say they will also spread to the rest or kill themselves once they are told they have* HIV” *(R5/FGD1, a 26-year-old donor and mother with 1 child).*

Another participant also noted

“*People don’t want to go for check-ups to know their HIV status that will hinder very many mothers from donating breast milk. And I feel if they were just taking breast milk without checkups the lines would have been very long*”. *(R6FGD2, 25 years old of 3 children).*

### Negative Cultural beliefs

Cultural beliefs and myths about human milk donation were cited as hindrances.

Some of the participants noted that women will be suspicious about where exactly they are taking their human milk, and ill-intentioned people may harm the donor through human milk donation.

“*Like some will be asking now they are asking for my breast milk and they take it where or do what with it?” (R5/FGD4, a 30-year-old mother of 3 children).*

The fears and cultural beliefs were compounded by the limited information about human milk donation.

*“They will be conservative. It is something they have never heard of; they will think they are going to kill women or make them never to produce children. That kind of stuff is likely to come up as a fear from traditional leaders. That kind of fear will be put in women, and they will fear donating milk”. (R5/FGD4, a 30-year-old mother of 3 children).*

Healthcare workers also mentioned that some mothers may fear transmission of criminality such as theft, murder and mental illnesses through the donor human milk, as one of them explained:

*“There are some genetic issues everything is to do with a DNA thing. Some people fear their babies to feed on the breasts of thieves, murderers, mentally disturbed people and the like that their children will end up like that. Then another thing as Africans we respect totems, in Ankole there is a clan where by their wives can’t breastfeed another person’s baby because if you do it the child will die. In Buganda I hear you don’t eat or move close to the clans or totems.” (KII1 NICU nurse, a mother and frequent human milk Donor at St. Francis Hospital Nsambya).*

### Partner/Spouse refusal

The other critical aspect that was brought up through the discussions was partner refusal for mothers to donate human milk for banking. Most women believed that their spouses would not agree to it. Some actually shared their experiences where their spouses refused them from donating as noted;

“*Mine was good and supported me, but others refuse their wives to donate their babies’ milk; it will be reduced, and it is not enough for their baby”. (KII4, frequent donor and staff at Nsambya Hospital).*

Another one added:

*“One time my hubby asked me, don’t you think you are depleting for our baby. And I would tell him let us share. He was hesitant first but later had to allow me. But then another friend of mine the husband refused her to give his son’s milk”. (Mother of one, and a frequent donor at Nsambya Hospital).*

However, it was noted that for some of the participants, their spouses were supportive of the idea of human milk donation, which was a great motivation for them.

*“My husband was good and supported me in donating breast milk to those who were in need of it”. (KII4, frequent breast milk donor at Nsambya Hospital).*

### Suggestions to improve accepting to donate

Throughout the discussions and interactions with the participants, a number of suggestions were made on how to overcome the barriers and get women to donate human milk to those who are in need.

### Saving time for the donors by collecting human milk from home or workplace

Throughout the discussions, the aspect of time was very critical, as most participants wondered where they would get the time to keep going to donate human milk since most of the potential donors are working and have other obligations to fulfil. Participants expressed the need for people to collect milk from their homes or places of work.

*“How I wish there will be some people who will keep passing through to collect donated breast milk from the willing mothers. I think people should endeavor to find us at our places of work or homes”. (R6/FGD2, a 25-year-old mother of 3 children).*

### Doctors to assess those suitable to donate

The participants felt that the health workers had an important role to play in getting mothers accept to donate human milk. They noted that health professionals were better placed to explain to potential donors the importance of donating human milk and getting women to participate. They also felt that the health professionals needed to use their expertise to assess those who qualify to donate and those who don’t as one of the participants noted.

“*So doctors need to assess well whether the mothers have enough for their babies and then donate like they do during blood donation”. (R3/FGD1, a 33-year-old mother with 3 children).*

### Screening human milk donors for infections

Participants noted that some mothers may refuse the donated milk for fear of passing on infections to their children. They suggested thorough screening of the milk so that the mothers of the preterm infants could have confidence to give their babies that milk.

*“But I request the doctors to test so well, the donors to see that the milk is so free from any diseases. HIV has an incubation process how we know that the milk is so free from HIV. Let us know within what duration should a woman be tested again? Because at any time one can acquire a disease. So I pray and request that the milk be well scrutinized before it is taken to the bank to save the lives of the preterm”. (R2/FGD3, 28-year-old mother).*

### Community education about human milk donation

Participants noted that many people are not aware of the possibility of human milk donation. People have many beliefs about it, yet there are thousands of potential human milk donors. They further noted that when people are sensitized about the importance of human milk donation, they will do it.

*“I think ladies should be taught about it and they will be willing to donate it is about awareness creation. They need to know that some mothers lack breast milk or that some preterm infants have lost their mothers. When they learn and see what happens in the NICU, then they will be kind to donate”. (R6/FGD2, 25-year-old mother of 3 children).*

“*Awareness creation is very important to these ladies now like my other baby I had a lot of breast milk, and I would just waste it. I didn’t know that some people look for breast milk for their preterm when they don’t have. I could leave it to pour so that it reduces had I know I would have come and donate”. (R5/FGD4, a 30-year-old mother of 3 children).*

### Involvement of community leaders

It was noted from the discussions that the involvement of community leaders and elderly people would facilitate the acceptance of human milk donation. Participants noted that if the community leaders and elders understood the idea and its importance, they would influence the women to do it. Some participants noted that community leaders/elders were not conversant with formula feeds; they knew about human milk and its importance. These were considered to be facilitators if sensitized.

*“Some might be young and might not understand these issues well, but traditional leaders I think will encourage us to donate some breast milk. They know the value of breast milk and always encourage us to breastfeed our babies longer. So I feel what they need is sensitization and have information about donation. They will support the idea”. (R8/FGD1, 23 years old, a mother of 5 children).*

*“.. we need to train the traditional and community leaders about breast milk donation. What they know most of is wet nursing, so we need to sensitize them and de-campaign that exercise of another mother breastfeeding another’s baby and promote donation of breast milk.” (R4/FGD1, 23-year-old first-time mother).*

One of the participants shared her experience on the role of her grandmother during her experience with a premature

*“I think the community leaders will buy the idea because traditional leaders know less of formulas, so they know that breastfeeding is on point for babies. I had a premature baby at 7 months, and I had no milk. They called my granny and they told her about it she told them that they should call my aunt and she gets tested and she helps me to get breast milk for my baby”. (R2/FGD1, 29 years old, mother of 2 children).*

## Discussion

Our study sought to determine the level of acceptance to donate human milk and assessed factors associated with mothers’ acceptance to donate human milk. Over three quarters of mothers were willing to donate human milk. Hence, the acceptance to donate human milk was high. This high level of acceptance correlated with the positive perception of human milk as useful, awareness about wet nursing and visiting under the five OPDs. This could be because mothers at the hospital had been exposed to human milk donation. In the past five years, the neonatal unit at St. Francis Hospital Nsambya has improvised pairing mothers who are breastfeeding with mothers who have very low birthweight preterm babies as human milk donors. In line with promoting human milk donation for babies in need and laying ground for the establishment of the human milk bank. Similar studies that found high acceptance rates were conducted in Turkey (64%) and India (84.9%) [23, 24]. Similar to India and Turkey, the study results are most likely influenced by the availability of information about the need for human milk banks in the care of premature babies shared through various forums. Our results (77.6%) are better than those observed in Nigerian and Ethiopian studies, in which acceptance was 11% and 39% [23, 24], respectively. The low levels of accepting to donate human milk were linked to lack of awareness about human milk banking and mother’s fears and traditional myths associated with human milk banking that are prevalent in the community [16].

In this study, factors associated with the acceptance to donate human milk were positive towards human milk expression and storage, knowledge and awareness about wet nursing and visiting under-five OPDs.

Mothers who had knowledge and were aware of wet nursing were 2.3 times more likely to donate human milk than were mothers without wet nursing knowledge and awareness. It is likely that the practice of wet nursing exists in communities, and mothers with knowledge about this wet nursing practice have an increased tendency to accept donating human milk to children in need. This was also supported by qualitative findings that mothers who had seen babies grow on other women’s milk (wet nursing) were supportive of human milk donation. The experience with wet nursing increased women’s confidence that they can perform the practice [25]. in line with what Sekhon describes as self-efficacy [18]. Similarly, mothers who heard about wet nursing in Ethiopia were more likely AOR 4.2 (95% CI 2.5, 6.9) to accept human milk donation [10]. In Brazil, it was also found that awareness about donor human milk banking and wet nursing were significantly associated with acceptance to donate human milk for banking [26]. In our study, awareness of human milk banks was not significant, possibly because there is currently no human milk bank in Uganda.

According to our study findings, mothers who visited the under-five OPD were 21 (AOR 95% CI 2.30, 196.9) times more likely to accept donating their human milk. This is most likely because in our setting, information about breastfeeding newborns is mostly provided during antenatal, postnatal and at the under-five outpatient departments. Whereas our findings indicate a wide confidence interval, they are similar to those observed in Ethiopia, in which mothers were twice as likely to donate their human milk after visiting a children’s ward [10]. Visiting units will expose mothers to knowledge about breastfeeding. It may be that as mothers receive information related to breastfeeding and the importance of human milk to the infants, their acceptance to donate human milk increases.

In our study, mothers who thought collecting and storing human milk was useful were 7.5 (95% CI 3.0, − 18.6) times more likely to accept donating their breast milk. This can be partly explained by the fact that working-class mothers who practice expression of human milk for their own babies have no objection to donating their excess milk to another baby. This was comparable to a study conducted in Brazil that showed that information about breast milk expression was significantly associated with human milk donation AOR 3.6 (95% CI 1.48, 8.9) [26].

In our study, we found the most common reasons for accepting to donate human milk among postnatal mothers were having excess breast milk (66%), followed by helping infants (63%) in need and supporting mothers who lack human milk (55%). This is supported by findings from the FGDs and KIIs, where most of the participants were willing to donate, especially because the preterm infants are unlikely to survive without human milk and they considered human milk donation to be an act of kindness, and a sacrifice to save a life. Most participants urged that there was no substitute for human milk for a newborn baby, whether preterm or full term. They noted that mothers who did not breastfeed their babies were denying them of the best quality food and that it would interfere with the proper development of their children. Some of the mothers also considered human milk donation as a way of reducing congestion of the breast; they looked at it as a relief for the mother who is experiencing painful breasts due to milk engorgement. However, expressing human milk while engorged should not be encouraged as it will worsen the problem. A qualitative study done in Eastern Uganda revealed that knowledge and recognition that human milk is better than formula milk by most study participants facilitated acceptability of donor human milk [25]. In line with [18] acceptability framework, our participants were generally positive about human milk donation, felt it is feasible to undertake and are effective for newborn care, especially for premature infants.

The most common barriers to accepting human milk donation among participants were fears of not having enough milk, not having time for the donation process, stress affecting milk flow, fear of transmitting diseases, knowing one’s HIV status, and loss of body image secondary to breast milk expression and donation. There were also negative cultural beliefs, such as a belief that criminal behaviours and mental illness can be transmitted to the baby through human milk if the donor happens to have such. Sekhon et al. stated that acceptability of healthcare interventions depends on the appropriateness of the intervention in terms of anticipated or experienced cognitive and emotional responses [18]. They stated 7 constructs for the theoretical frame work of acceptability (TFAs), which include affective attitude (how an individual feels about the intervention), burden (perceived effort required to participate in the intervention), ethicality (intervention’s goodness of fit with individual’s value system), intervention coherence (participant’s understanding of how intervention works), opportunity costs (benefits, profits and values to be given up to engage in the intervention), perceived effectiveness (perception that intervention has achieved purpose), and self-efficacy (participant’s confidence that they can perform the intervention). Our participants generally felt good about human milk donation but may be burdened by the time spent in the process of human milk donation. This finding is not new and has been reported in another study in Hong Kong [27], where mothers mentioned time as a major obstacle to human milk donation. Plans to start human milk banks should incorporate strategies to ensure that the process of donating is devoid of delays. Some participants suggested considering home and workplace pick-up of human milk. Most study participants were confident of the effectiveness of human milk donation, especially those who had donated before and those who had seen babies survive and grow on donated human milk, in line with the construct of perceived effectiveness in the acceptability framework [18]. Most mothers perceived human milk to be effective in preventing disease and improving children’s immunity and mental and physical development [28]. Mothers who had sufficient human milk viewed donating excess milk as valuable to save babies of mothers without human milk. In contrast, there was fear among most study participants that donating human milk may deprive their own babies of adequate nutrition.

This requires creating awareness among donor/recipient and also for health care workers. Healthcare officials have the added responsibility of assessing and advising mothers suitable for human milk donation. The concern that mothers may not be having enough milk for their own babies to donate has also been raised in some studies [16, 27]. Our study revealed that negative cultural beliefs such as criminal behaviours and illness can be transmitted to the baby through donated human milk, reflecting value conflict in acceptance to donate human milk and requiring community education and dialogue to counter. In addition, some mothers were concerned that some male partners would not approve human milk donation for fear that it would deprive their babies of adequate milk. This finding is not surprising given that men in many African settings are key decision makers on issues related to child and maternal health [29]. It is important that health care professionals target community leaders and male partners as key stakeholders in health education regarding human milk donation and decision making. Our findings also revealed a misconception that breastfeeding and human milk donation may lead to sagging of breasts which may make women unattractive to men as has been documented in Uganda [30] and other African settings [31–33]. Efforts to promote breastfeeding and human milk donation should encourage mothers to breastfeed and reassure them that breastfeeding does not alter breast appearance [34, 35].

### Strength/limitations

To our knowledge, this was the first study to assess the acceptability of human milk donation in Uganda at the time when St. Francis Hospital Nsambya is planning to start a human milk bank. The study had quantitative and qualitative component findings that complemented each other in understanding the acceptability of human milk donation and factors at play. In the qualitative part, we obtained data from mothers and health care workers and thus provided an opportunity for data triangulation, which improved the trustworthiness of our study findings. The study was conducted in a hospital that is a private non-profit. Most postnatal mothers attending the hospital have better access to health education and income than ordinary Ugandans. Thus, the results may not be generalizable to other Ugandan settings. In addition, the high acceptability documented in our setting may be attributed to the exposure of mothers to human milk donation, which is a practice to feed very low birthweight preterm at St. Francis Hospital Nsambya using locally improvised methods. At the time of the study, St. Francis Hospital Nsambya was conducting community education in preparation to start a human milk bank, which is not the case in other Ugandan settings. Recall bias cannot be excluded in the interview process; however, our study participants were those within six weeks of delivery, which helped to minimize the effect of recall bias. This was a hospital-based study; thus, the views of mothers outside the hospital setting about human milk donation may vary. We recommend community-level studies involving all stakeholders, including community leaders, elders and spouses/partners.

## Conclusions

The level of acceptance to donate human milk among mothers at St. Francis Hospital Nsambya at 77.6% is high. Thus, establishing a human milk bank is feasible in the study setting. Being positive for breast milk expression and storage, awareness of wet nursing and visiting an under-five-OPD were associated with acceptance of human milk donation.

Human milk is generally considered the best food for infants, especially premature babies. Key barriers to accepting human milk donation were fear of delays during human milk donation, not having enough milk, spousal refusal and a belief that criminal behaviours and mental illness can be transmitted through donated human milk. Making the process of donation quick, picking up services for donors, and community education and male partner engagement in human milk donation could further increase the acceptability of human milk donation. The information obtained will guide messages for the particular women in the postnatal wards. The stakeholders can use the information to set up a human milk bank at St Francis Hospital Nsambya.

## Data Availability

The datasets generated during and/or analysed are available from the corresponding author on reasonable request.

## Abbreviations

ANC: Antenatal Care
DHM: Donated Human Milk
ELBW: Extremely Low Birthweight
FGD: Focus Group Discussion
HIV: Human Immunodeficiency Virus
HBMB: Breast Human Milk Bank
IPD: In Patient Department
IQR: Interquartile Range
KII: Key Informant Interview
LBW: Low Birthweight
NICU: Neonatal Intensive Care Unit
OPD: Out Patient Department
PDHM: Pasteurized Donated Human Milk
PNC: Postnatal Care
ROP: Retinopathy of Prematurity
SFHN: St. Francis Hospital Nsambya
TFA: Theoretical Frame work of Acceptability
VLBW: Very Low Birthweight
WHO: World Health Organization
